# Supplementary material for: Using subject-level variability to predict time-varying outcomes: investigating the association between hormone variability and BMD trajectories over the menopausal transition
Source: J Appl Stat. 2025 Nov 26;53(11):2137–61. doi: 10.1080/02664763.2025.2589991 (PMC13537070; doi:10.1080/02664763.2025.2589991)
Supplement: Supplementary file 1 [file CJAS_A_2589991_SM0335.pdf]

Supplementary material: Using Subject-Level Variability to Predict Time-Varying Outcomes: Investigating the Association between Hormone Variability and BMD Trajectories over the Menopausal Transition

Irena Chen[1*], Zhenke Wu[2, 3], Sioban D. Harlow[4], Carrie A. Karvonen-Gutierrez[4], Michelle M. Hood[4], Michael R. Elliott[2,5]

[1*]Department of Digital and Computational Demography, Max Planck Institute for Demographic Research, Rostock, Germany.
[2]Department of Biostatistics, University of Michigan,Ann Arbor, Michigan, USA.
[3]Michigan Institute for Data and AI in Society (MIDAS), University of Michigan, Ann Arbor, Michigan, USA.
[4]Department of Epidemiology, University of Michigan, Ann Arbor, Michigan, USA.
[5]Department of Survey Methodology, University of Michigan, Ann Arbor, Michigan, USA.

*Corresponding author(s). E-mail(s): chen@demogr.mpg.de;

S1 Additional Figures

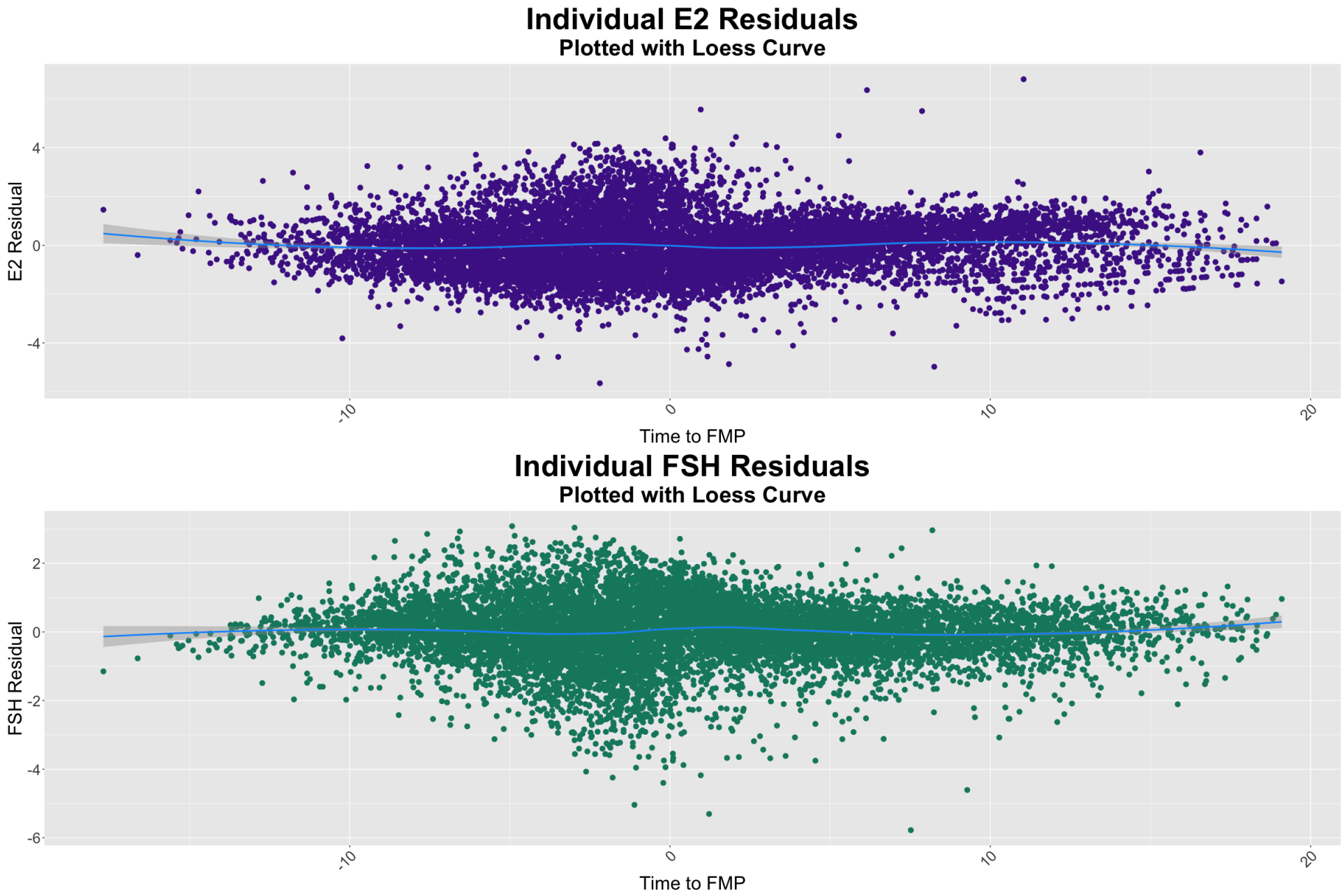

Fig. S1: Plot of E2 residuals (top figure) and FSH residuals (bottom figure) for all individuals in our dataset, plotted over time to FMP. A loess curve has been added to each plot to show the average population trend. Both E2 and FSH observations (before detrending) had been $\log_2$ transformed.

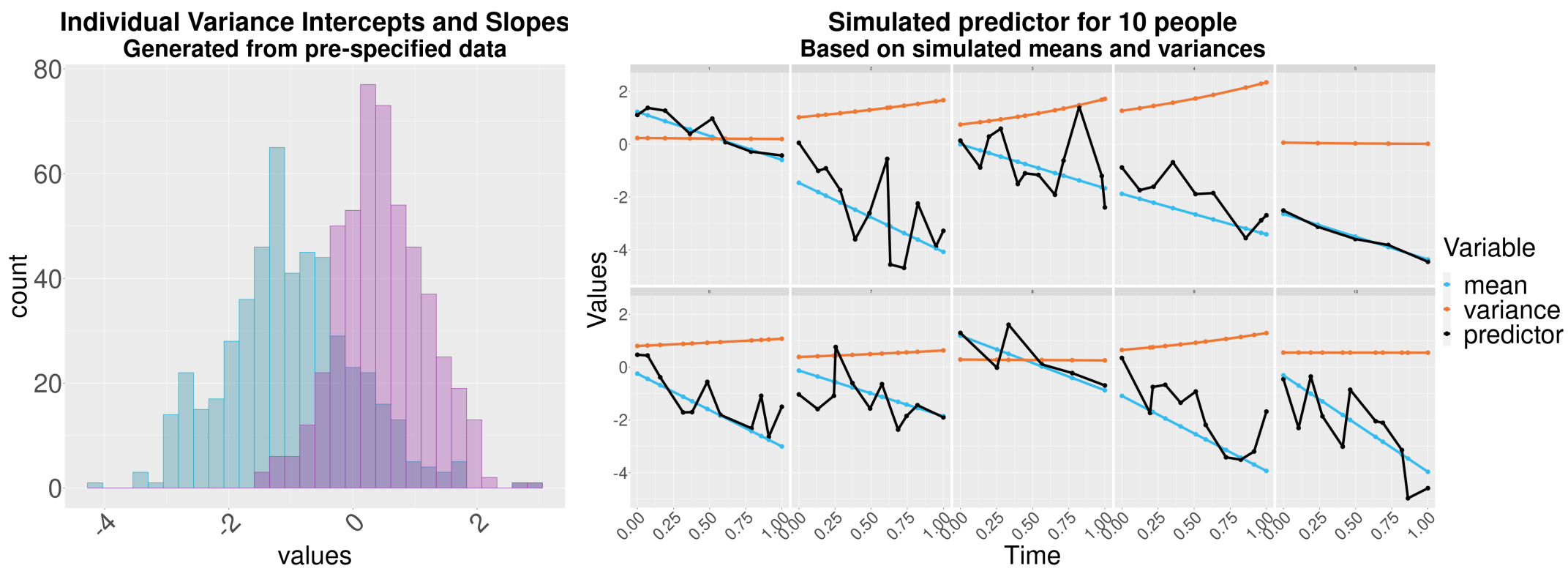

Fig. S2: Individual variance intercepts (left, flatter histogram) and slopes (right, peaked histogram) and individual mean, variance and marker trajectories (line plots) for the "high variability" simulation scenario in Simulation Study II. When the individual variability trajectory is increasing, we see that the marker trajectory has higher variability between measurements.

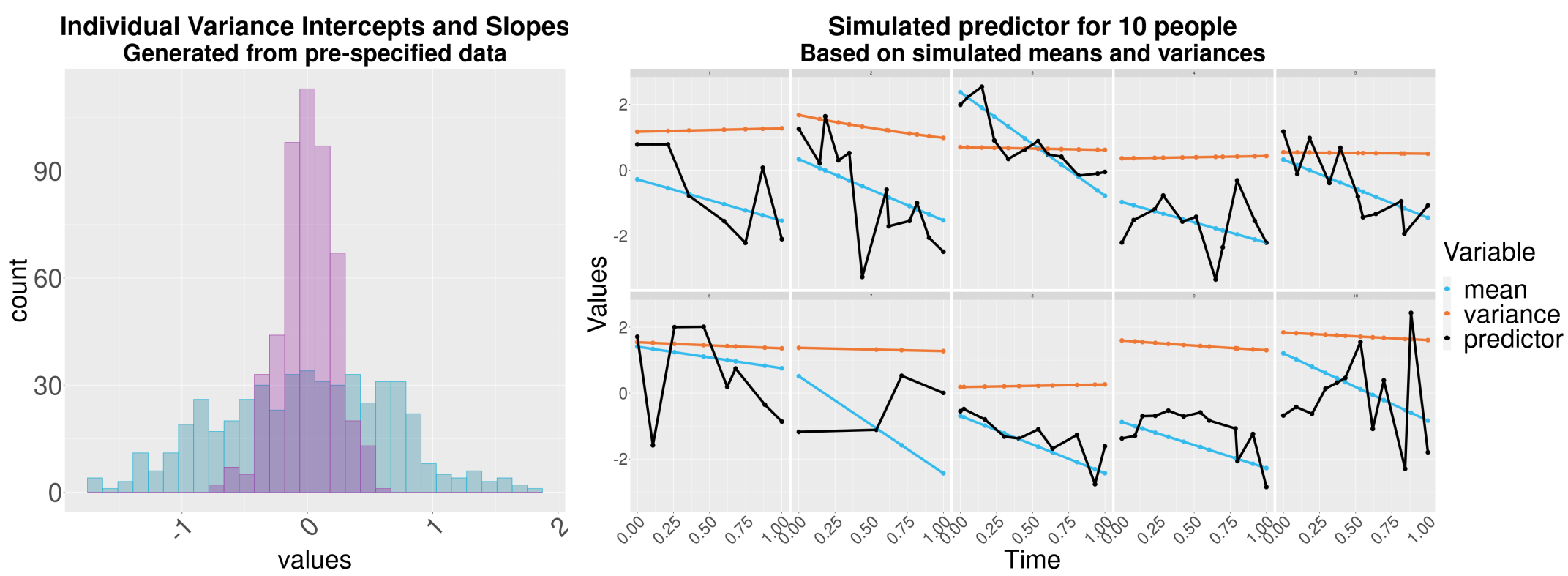

Fig. S3: Histograms (left plot) of individual intercepts (flatter histogram) and slopes (peaked histogram) and individual mean, variance and predictor trajectories (line plots) for the "low variability" simulation scenario in Simulation Study II.

S2 Posterior Predictive Model Checking

To assess our model's validity on the SWAN data, we conduct posterior predictive checks for both the predictor submodel and the outcome submodel.

For the outcome submodel, we generated simulated data from the posterior predictive distribution. The posterior predictive distribution for the predicted outcome,

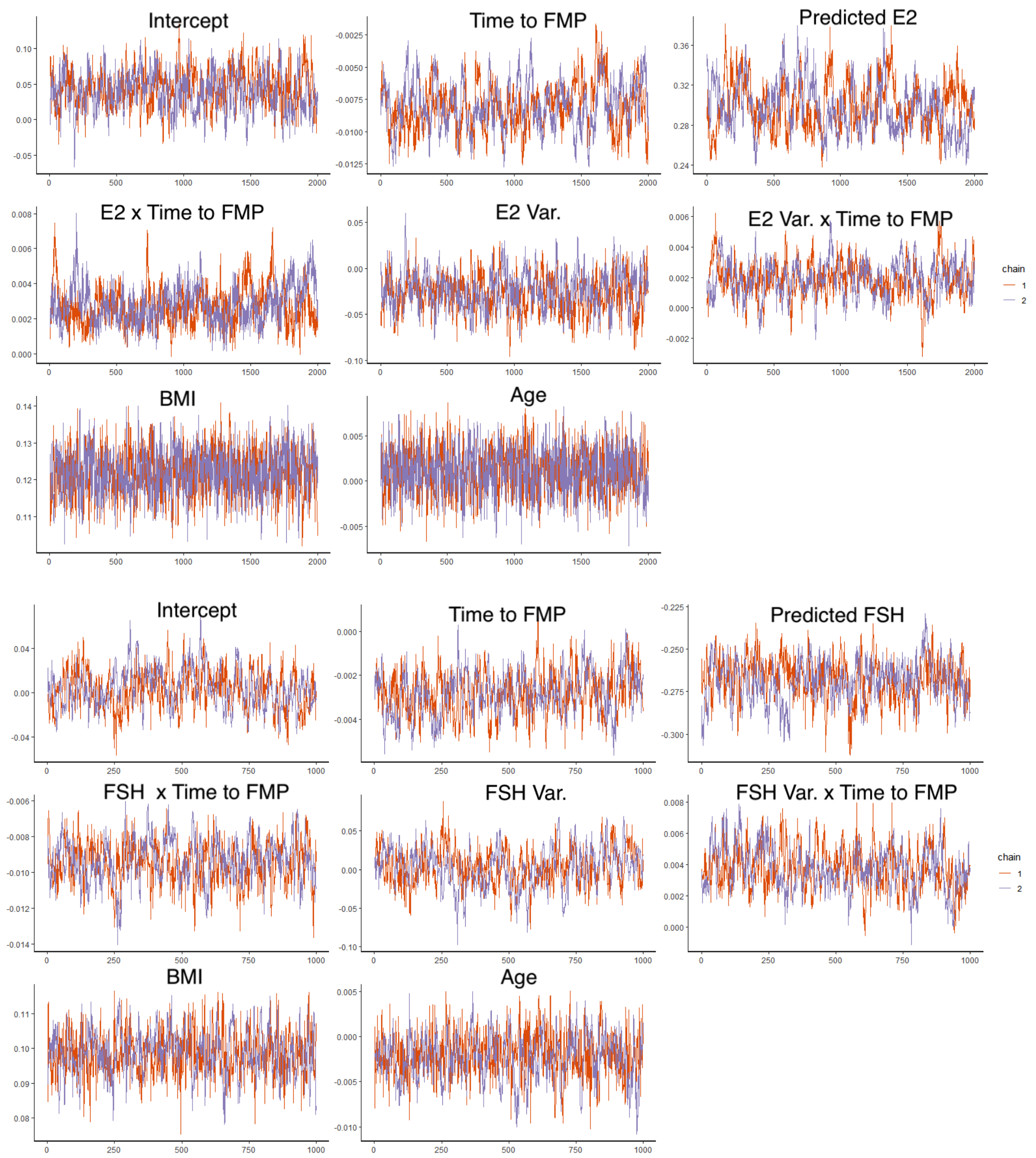

Fig. S4: Traceplots for the outcome parameters in the E2-BMD model (top figure) and in the FSH-BMD model (bottom figure). The estimated R-hat values for these parameters were < 1.05, which further supports that the chains had converged relative to the R-hat criterion < 1.05

$\widetilde{Y}$ can be written as:

$$p(\widetilde{Y}|Y) = \int p(\widetilde{Y}|\theta, X)p(\theta, X|Y)d\theta dX$$

where θ are the unknown model parameters and X are the predictor variables used in the outcome regression. For each draw of the model parameters from the posterior

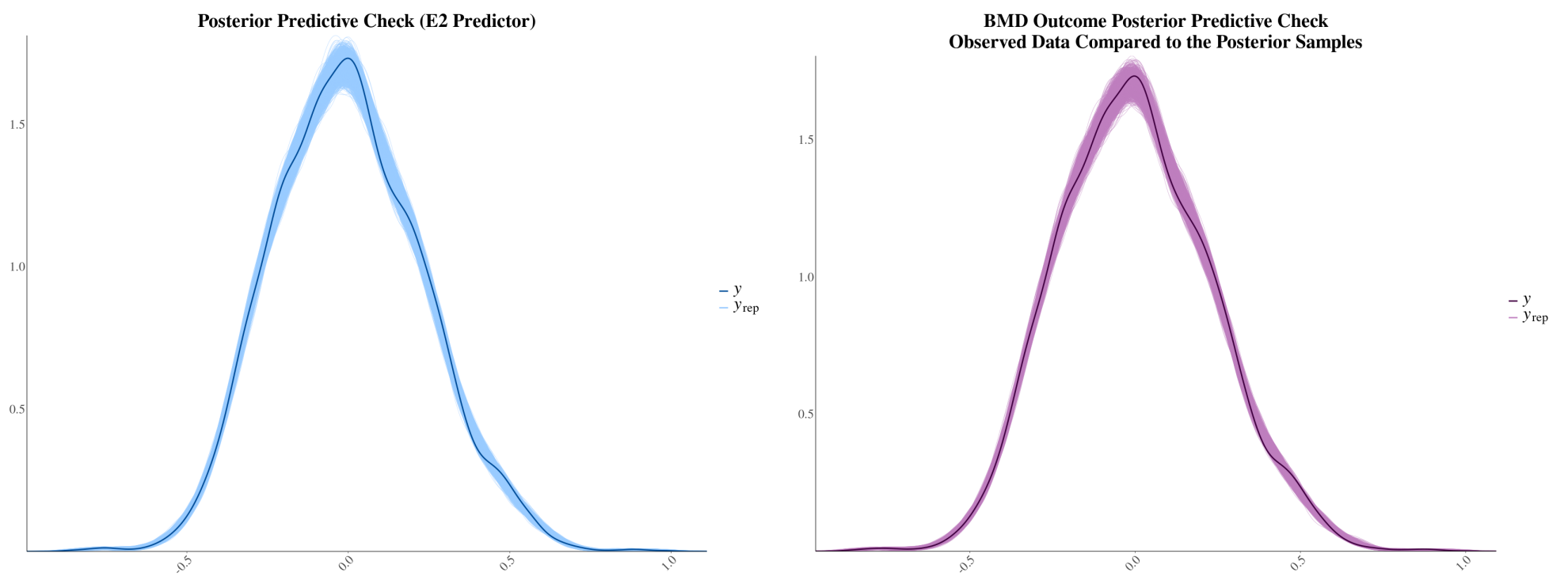

Fig. S5: Visualization of the posterior predictive checks performed for the E2 predictor model (left) and the FSH predictor model (right).

distribution, $p(\theta|Y, X)$, we can draw a vector $\widetilde{Y}$ from the posterior predictive distribution by conditioning on the draw of the model parameters and then simulating from the data model (Gabry et al, 2019).

We then plotted 1,000 draws of this model-generated data against the observed outcome, which is shown in Figure S5. The observed outcomes (y) are plotted with the solid lines and the model-generated outcomes (yrep) are plotted with the thin semi-opaque lines. These plots show that the model-generated outcomes cover the observed BMD residuals, indicating that our model is generating reasonable estimates of the outcomes. For both models, we see that the simulated replicated data from the model overlap the observed data, indicating that our model is producing reasonable predictions.

For the longitudinal biomarker predictor submodel, we define the following statistic:

$$T(x_{ij}; \boldsymbol{b}_i, t_{ij}) = \sum_{ij} (x_{ij} - \mu(\boldsymbol{b}_i, t_{ij}))^2 / \sigma_i,$$

where $\mu(\boldsymbol{b}_i, t_{ij}))$ is the i-th individual's mean biomarker trajectory and σ_i is the estimated standard deviation of individual i's biomarker trajectory. By doing this, we can compare $T_i(x_{ij}^{obs}; \boldsymbol{b}_i, t_{ij})$ (which is a function of the observed data and the estimated parameters) with $T_i(x_{ij}^{sim}; \boldsymbol{b}_i, t_{ij})$, which is a function of the model generated data using the model estimated parameters). If there are large discrepancies between $T_i(x_{ij}^{obs})$ and $T_i(x_{ij}^{sim})$, this could indicate poor model fit (Gelman et al, 2013).

One way to compare these two $T(x_{ij}; \boldsymbol{b}_i, t_{ij})$ statistics is to compute the 'posterior predictive p-value', which is

$$P(T_i(x_{ij}^{obs}; \boldsymbol{b}_i, t_{ij}, s_i^2) < T_i(x_{ij}^{sim}; \boldsymbol{b}_i, t_{ij}, s_i^2) | (x_{ij}^{obs})).$$

For E2 and FSH, we keep (x_{ij}^{obs}) fixed at the observed values and compute 1,000 values of $T_i(x_{ij}^{sim}; \boldsymbol{b}_i, t_{ij})$ from the posterior of $\boldsymbol{b}_i, t, s_i$. We then compare these values

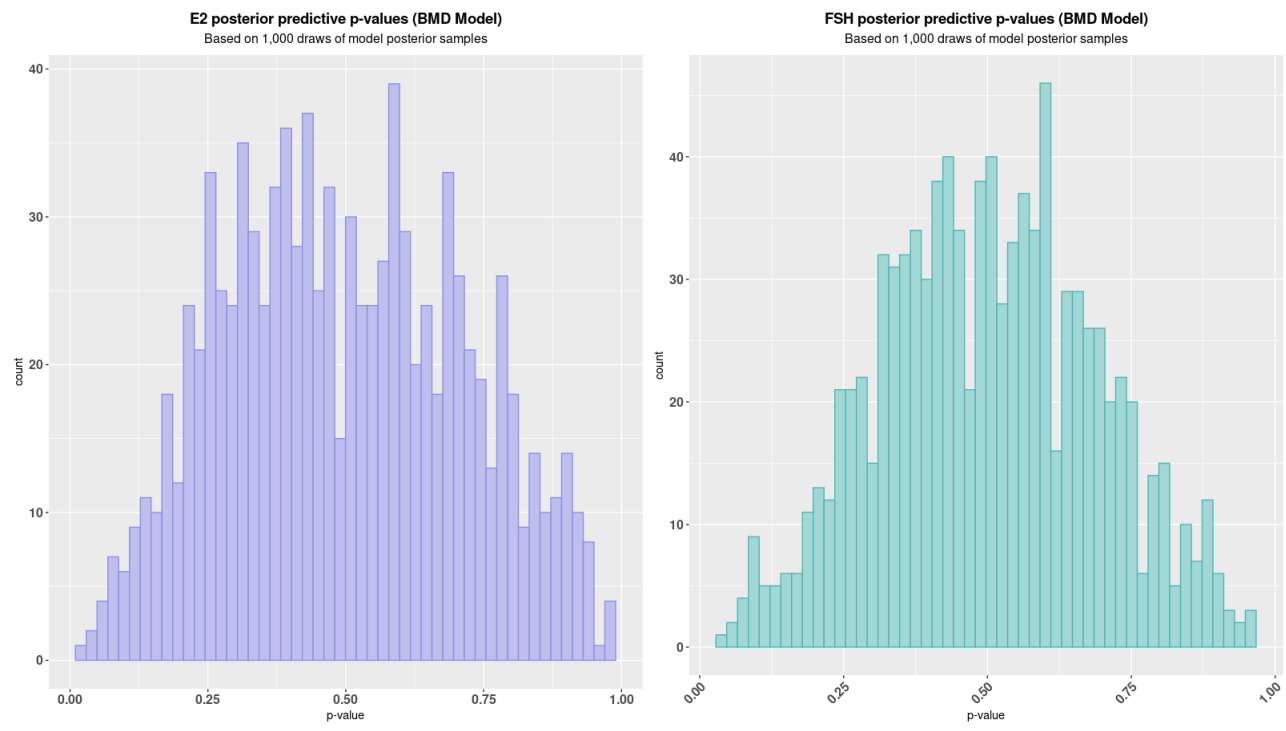

Fig. S6: Visualization of the computed p-values performed for the BMD outcome model. The distributions of the p-values indicate that our model was able to fit the hormone residual data reasonably well.

with 1,000 draws from $T_i(x_{ij}^{sim}; \boldsymbol{b}_i, t_{ij}, s_i)$. Figure S6 displays the histograms of the resulting p-values for each individual's E2 trajectory. The majority of the computed p-values were between 0.25 and 0.75. Further analysis of the p-values across the quantiles of the distribution shows that the generated data from the model reasonably captures the individual trends. This provides justification that both the predictor submodel and the outcome submodel are good fits for the data.

S3 Data Analysis: Study of Women's Health Across the Nation

S3.1 Additional Model Estimated Parameters

In this section, we present the estimated posterior means and 95% credible intervals for the other parameters from the BMD outcome models in Section 4 of the main text. Table S1 displays the estimated posterior means and 95% CrIs for the E2 predictor model and Table S2 displays the posterior means and 95% CrIs for the FSH predictor model. All R-hat values for these parameters were < 1.01, indicating the models had converged.

Parameter	Post. Mean	2.5% CrI	97.5% CrI
α_1	-0.03	-0.06	0.00
α_2	0.02	0.01	0.02
$\Sigma[1, 1]$	0.09	0.07	0.11
$\Sigma[1, 2]$	0.00	0.00	0.00
$\Sigma[2, 2]$	0.00	0.00	0.00
ν	0.16	0.11	0.20
ξ	0.38	0.34	0.43
σ	0.04	0.04	0.04
τ	0.19	0.18	0.19
β_0	0.04	-0.02	0.09

Table S1: Evaluation of the posterior means and 95% CrI estimates for the other parameters in the E2-BMD outcome model. All posterior means and 95% CrIs are presented in their original values.

Parameter	Post. Mean	2.5% CrI	97.5% CrI
α_1	0.02	-0.01	0.06
α_2	0.00	0.00	0.00
$\Sigma[1, 1]$	0.16	0.13	0.19
$\Sigma[1, 2]$	0.00	0.00	0.00
$\Sigma[2, 2]$	0.00	0.00	0.00
ν	-0.55	-0.61	-0.50
ξ	0.56	0.51	0.61
σ	0.04	0.04	0.04
τ	0.19	0.18	0.20
β_0	0.11	-3.54	3.77

Table S2: Evaluation of the posterior means and 95% CrI estimates for the other parameters in the FSH-BMD outcome model. All posterior means and 95% CrIs are presented in their original values.

Truth	Scenario	Model	Bias	Coverage (%)	Average Interval Length
β_0 = 2	HV	JELO	0.00	96.5	0.12
	HV	JELO (CV)	0.05	68.5	0.12
	LV	JELO	0.00	95.0	0.24
	LV	JELO (CV)	0.00	93.0	0.24
β_1 = -1.5	HV	JELO	0.00	91.0	0.08
	HV	JELO (CV)	0.00	93.5	0.08
	LV	JELO	0.00	92.5	0.10
	LV	JELO (CV)	0.00	91.0	0.10
β_2 = 0.25	HV	JELO	0.00	94.5	0.15
	HV	JELO (CV)	-0.20	0.5	0.11
	LV	JELO	0.00	95.0	0.19
	LV	JELO (CV)	0.00	95.4	0.19
β_3 = 1	HV	JELO	0.00	98.5	0.18
	HV	JELO (CV)	-0.05	82.5	0.18
	LV	JELO	-0.01	97.5	0.06
	LV	JELO (CV)	0.00	97.0	0.06
β_4 =0.75	HV	JELO	0.00	95.5	0.05
	HV	JELO (CV)	0.01	82.5	0.05
	LV	JELO	0.00	95.0	0.27
	LV	JELO (CV)	-0.01	96.4	0.26
β_5 = -0.10	HV	JELO	0.00	96.0	0.12
	HV	JELO (CV)	0.29	0.0	0.10
	LV	JELO	0.00	99.0	0.06
	LV	JELO (CV)	0.01	96.4	0.13

Table S3: Simulation II: bias, coverage, and 95% credible interval length across 200 simulation replicates for each data scenario (CV = constant variance)

S4 Simulation II: Full table of simulation results

S5 Simulation III: Varying number of individuals and timepoints

S5.1 Constant variance scenario with fewer individuals and more timepoints

Additionally, we performed another set of simulations where we varied the number of individuals and observations per individual. In the first set of simulations, we set the number of individuals to 100, a decrease from Simulations I and II. In the second set of simulations, we kept $I = 100$, but increased the number of observations for each individual to be between 20-50, instead of 3-12. The results of the simulations for the outcome parameters is shown in Table S4. Interestingly, we see that the coverage for β_3, β_5 (the variance parameters) for $I = 100$ individuals with *more* timepoints is lower, compared to when the same number of individuals have more observations. Additionally, the bias of β_3, β_5 are both towards 0, indicating that the effects of the variances (both unadjusted and interacted with time) are being underestimated. The average interval lengths in the scenario with fewer observations per individual is higher

than in the scenario with more observations are higher, which correctly indicates the higher uncertainty that comes with the smaller dataset.

This result suggests that if the number of individuals is small, an increase in the number of observations per individual may not necessarily result in reliable or accurate estimates of the variances.

Truth	Scenario	Bias	Coverage (%)	Average Interval Length
β_0 = 2	I = 100	0.00	88.5	0.66
	I = 100, More Timepoints	0.14	73.4	0.40
β_1 = -1.5	I = 100	-0.06	95.0	0.61
	I = 100, More Timepoints	0.13	91.0	0.33
β_2 = 0.25	I = 100	0.00	94.0	0.26
	I = 100, More Timepoints	0.00	92.0	0.06
β_3 = 1	I = 100	-0.14	74.0	0.50
	I = 100, More Timepoints	-0.15	39.5	0.26
β_4 =0.75	I = 100	0.00	96.0	0.13
	I = 100, More Timepoints	0.00	95.5	-0.06
β_5 = -0.10	I = 100	0.06	87.5	0.31
	I = 100, More Timepoints	0.05	59.3	0.13

Table S4: Simulation : bias, coverage, and 95% credible interval length across 200 simulation replicates with a) Fewer timepoints (3-12 observations per individual) and b) More timepoints (20-50 per individual). Each replicate simulated data for 100 individuals.

S5.1.1 Time-varying variance scenarios with fewer individuals and more timepoints

We also examined the performance of the time-varying variance model under different scenarios with different numbers of individuals and timepoints. In the first scenario comparison, we set $I = 200$ and allow each individual to have between 3-12 observations. For the second comparison, we keep $I = 200$ and allow each individual to have between 20-50 observations. We do this for both the HV (high-variance) and LV (low-variance) settings; details of these two settings can be found in Simulation Study II in the main text. Table S5 shows the bias, coverage, and average interval lengths of each of the 4 different scenarios. The bias for all outcome regression coefficients remained low across all of the scenarios, and the coverage for all of the coefficients was consistently high. For β_3, β_5, the coefficients corresponding to the individual variances, we do see that the HV scnenario with fewer individuals and fewer observations has wider average interval lengths than when we increase the number of individuals. This suggests that when the individual variances are systemically changing across time, increasing the number of observations per individual may result in reduced uncertainty when estimating the parameters.

Truth	Scenario	Model	Bias	Coverage (%)	Average Interval Length
$\beta_0 = 2$	HV	$I = 200$	-0.01	94.0	0.20
	HV	$I = 200$, More Timepoints	0.00	95.0	0.12
	LV	$I = 200$	-0.03	92.5	0.40
	LV	$I = 200$, More Timepoints	0.00	96.0	0.21
$\beta_1 =$ -1.5	HV	$I = 200$	0.00	92.4	0.30
	HV	$I = 200$, More Timepoints	0.00	95.0	0.17
	LV	$I = 200$	0.00	93.0	0.43
	LV	$I = 200$, More Timepoints	0.00	94.5	0.23
$\beta_2 = 0.25$	HV	$I = 200$	0.00	94.4	0.12
	HV	$I = 200$, More Timepoints	0.00	95.0	0.07
	LV	$I = 200$	0.00	95.5	0.17
	LV	$I = 200$, More Timepoints	0.00	96.0	0.09
$\beta_3 = 1$	HV	$I = 200$	0.00	94.4	0.25
	HV	$I = 200$, More Timepoints	0.00	97.0	0.14
	LV	$I = 200$	0.00	95.5	0.16
	LV	$I = 200$, More Timepoints	0.00	97.0	0.06
β_4 =0.75	HV	$I = 200$	-0.01	92.0	0.08
	HV	$I = 200$, More Timepoints	0.00	96.0	0.04
	LV	$I = 200$	0.00	93.5	0.10
	LV	$I = 200$, More Timepoints	-0.01	96.5	0.05
$\beta_5 =$ -0.10	HV	$I = 200$	0.00	91.0	0.21
	HV	$I = 200$, More Timepoints	0.00	96.5	0.10
	LV	$I = 200$	0.00	96.5	0.12
	LV	$I = 200$, More Timepoints	0.01	96.4	0.13

Table S5: Simulation II: bias, coverage, and 95% credible interval length across 200 simulation replicates for each data scenario.

S6 Simulation Studies: Additional Parameters

This section contains the bias, coverage, and average interval length statistics for the other JELO model parameters. For each simulation replicate, we ran two chains with 2,000 steps and 1,000 burn in. The data generation parameters are detailed in Section of the main text. Table S6 contains the estimates for the constant variance simulation study and Tables S7 and S8 contains the estimates for our time-varying variance simulation study.

Parameter	Truth	Average Post. Mean	Bias	Coverage %	Average Interval Length
α_1	0	0.00	0.00	95.5	0.14
α_2	2	2.00	0.00	95.0	0.13
$= \Sigma[1,1]$	1	1.00	0.00	95.0	0.22
$\Sigma[1,2]$	-0.05	-0.05	0.00	96.0	0.14
$\Sigma[2,2]$	1.01	1.00	0.01	94.0	0.19
ν	0	0.00	0.00	95.5	0.06
ξ	0.38	0.37	0.00	95.0	0.05
τ	1	1.01	0.01	96.0	0.01
σ	5	5.06	0.06	96.0	1.32

Table S6: Simulation Study 1: Evaluation of the JELO model bias, coverage, and 95% credible interval length across 200 simulation replicates.

Parameter	Truth	Bias	Coverage %	Average Interval Length
α_1	0	0.00	91.0	0.19
α_2	-2	0.00	95.0	0.17
α_{s1}	0	0.00	95.5	0.14
α_{s2}	2	0.00	95.0	0.13
$\Sigma[1,1]$	1	0.00	96.0	0.26
$\Sigma[1,2]$	-0.25	0.00	93.0	0.14
$\Sigma[2,2]$	0.5	0.00	93.5	0.14
$\Sigma_S[1,1]$	1	0.00	93.0	0.47
$\Sigma_S[1,2]$	0.1	-0.01	93.0	0.37
$\Sigma_S[2,2]$	0.5	0.00	95.0	0.52
τ	0.25	0.00	96.0	0.07
σ	0.1	0.00	92.0	0.01

Table S7: Simulation Study 2 (High Variance Setting): Evaluation of the JELO (with time-varying variance) model bias, coverage, and 95% credible interval length across 200 simulation replicates.

S7 Hamiltonian Monte Carlo

Since we used the Stan programming language and the `rstan` package to carry out HMC sampling, we clarify that the following description of the HMC algorithm is generic and are mainly summarized from the following excellent resources: Chapter 5 of (Neal, 2011), Betancourt (2018), and Homan and Gelman (2014).

Parameter	Truth	Bias	Coverage %	Average Interval Length
α_1	0	0.00	91.0	0.20
α_2	-2	0.00	93.0	0.20
α_{s1}	0	0.00	95.5	0.14
α_{s2}	0	0.00	95.0	0.13
$\Sigma[1,1]$	1	0.00	95.5	0.28
$\Sigma[1,2]$	-0.25	0.00	92.0	0.15
$\Sigma[2,2]$	0.5	0.00	93.5	0.15
$\Sigma_S[1,1]$	0.5	0.01	96.0	0.28
$\Sigma_S[1,2]$	-0.01	0.02	93.0	0.22
$\Sigma_S[2,2]$	0.05	-0.03	79.5	0.20
τ	0.25	0.00	93.0	0.10
σ	0.1	0.00	92.5	0.01

Table S8: Simulation Study 2 (Low Variance Setting): Evaluation of the JELO (with time-varying variance) model bias, coverage, and 95% credible interval length across 200 simulation replicates.

S7.1 HMC Dynamics

The HMC sampler generates proposals by using ideas from statistical mechanics to explore the posterior distribution (Hitchcock, 2003).

Let q represent a vector of position variables, and r a vector of "momentum" variables. In statistical terms, we can let q be the model parameters to be estimated. Our main target of interest, the posterior distribution of the unknown model parameters, is related to Hamiltonian dynamics via the following: $-\log \pi(q) = -\log [\ \pi_0(q)L(q|D)] \ = U(q)$ where π_0 is the prior distribution of the parameters q, $L(q|D)$ is the likelihood function given data D, and $U(q)$ is the "potential energy" function.

The Hamiltonian function is the sum of two energies: $H(r, q) = V(r) + U(q)$, where $V(r)$ represents the "kinetic energy". This is defined in (Neal, 2011) as: $V(r) = r^\mathsf{T} M^{-1} r/2$, where M is a "symmetric, positive definite mass matrix, which is typically diagonal and is often a scalar multiple of the identity matrix." $V(r)$ then corresponds to the (log) probability density of a Gaussian with mean zero and covariance matrix M.

In order to update r, q, the momentum and the model parameters, the algorithm simulates Hamiltonian dynamics using the Hamiltonian equations:

$$\frac{dq}{dt} = \frac{\partial H}{\partial r} = \frac{\partial U}{\partial r};$$
$$\frac{dr}{dt} = -\frac{\partial H}{\partial q} = -\frac{\partial U}{\partial r} - \frac{\partial V}{\partial q},$$

where $\frac{\partial V}{\partial q}$ is the gradient of the (log) target density. To solve these equations equations, we need a function that defines a path for (q, r) from which we can sample values of q.

S7.2 HMC Algorithm

To implement HMC, we need to define a few additional parameters. Let M be the number of samples we wish to draw using HMC (for example, in our applications, we set $M = 4,000$), and L be the number of leapfrog steps. Let ϵ be the step size. In Stan, the user can select the target acceptance parameter $\delta \in [0, 1]$, with a value closer to 1 indicating a smaller step size. A brief sketch of the HMC algorithm given in Neal (2011) is reproduced in S1, and described below.

The first step of the HMC algorithm is to propose new values for the momentum variables r (using their Gaussian distribution), independently of the current values of the position values. Once the momentum variables have been generated, apply leapfrog (usually the Newton-Störmer-Verlet integrator; see pages 119-122 of Neal (2011) for more details) updates to q, r. This will generate a proposal (q^*, r^*).

The second step uses Hamiltoninan dynamics to propose a new state, using the specified steps L and stepsize ϵ. The proposed state (q^*, r^*) is accepted with probability:

$$min\,[\,1, \exp(-H(q^*, r^*) + H(q, r))] \;= min\,[\,1, \exp(-U(q^*) + U(q) - V(r^*) + V(r))]\;,$$

which is the classic Metropolis-Hastings update. HMC ensures that the proposal distribution is symmetric by reversing the sign of r after each step, e.g.: $r^m = -r^m$ on the m-th iteration.

As noted in Betancourt (2018), the performance of the HMC algorithm is highly dependent on the choices of ϵ, L. The HMC algorithm implemented in `stan` uses the “no-U turn sampler”, which eliminates the need to select L.

References

Betancourt M (2018) A conceptual introduction to hamiltonian monte carlo. 1701.02434

Gabry J, Simpson D, Vehtari A, et al (2019) Visualization in bayesian workflow. Journal of Royal Statistical Society: Series A 182:389–402

Gelman A, Carlin JB, Stern HS, et al (2013) Bayesian Data Analysis. CRC Press

Hitchcock DB (2003) A history of the metropolis-hastings algorithm. The American Statistician 57(4):254–257. URL https://www.jstor.org/stable/30037292, publisher: [American Statistical Association, Taylor & Francis, Ltd.]

Homan MD, Gelman A (2014) The no-u-turn sampler: adaptively setting path lengths in hamiltonian monte carlo. The Journal of Machine Learning Research 15(1):1593–1623

Neal R (2011) MCMC Using Hamiltonian Dynamics, vol 20116022. Chapman & Hall/CRC, https://doi.org/10.1201/b10905-6

Algorithm S1 A generic HMC algorithm implemented with R code, originally from Neal (2011) and reproduced here with modifications.

```
        HMC = function (U, grad_U, epsilon, L, current_q)
{
q = current_q
r = rnorm(length(q),0,1) # independent standard normal variates
current_r = r

# Make a half step for momentum at the beginning
rp =r  - epsilon * grad_U(q) / 2

# Alternate full steps for position and momentum

for (i in 1:L)
{
# Make a full step for the position
q = q + epsilon * r
# Make a full step for the momentum, except at end of trajectory
if (i!=L) r= r - epsilon * grad_U(q)
}

# Make a half step for momentum at the end.
r = r - epsilon * grad_U(q) / 2
# Negate momentum at end of trajectory to make the proposal symmetric
r = -r

# Evaluate potential and kinetic energies at start and end of trajectory

current_U = U(current_q)
current_V = sum(current_r^2) / 2
proposed_U = U(q)
proposed_V = sum(r^2) / 2

# Accept or reject the state at end of trajectory, returning either
# the position at the end of the trajectory or the initial position

if (runif(1) < exp(current_U-proposed_U+current_V-proposed_V))
{
return (q) # accept
}
else
{
return (current_q) # reject
}
}
```
